# A Kenyan newspaper analysis of the limitations of voluntary medical male circumcision and the importance of sustained condom use

**DOI:** 10.1186/1471-2458-12-465

**Published:** 2012-06-21

**Authors:** Charlene N Muzyka, Laura H Thompson, Andrea E Bombak, S Michelle Driedger, Robert Lorway

**Affiliations:** 1 Department of Community Health Sciences, University of Manitoba, Winnipeg, Canada; 2 Centre for Global Public Health, University of Manitoba, Winnipeg, Canada

**Keywords:** Sexually transmitted infections, (East) Africa, HIV prevention, Media analysis, Male circumcision (MC)

## Abstract

**Background:**

Since the completion of three clinical trials indicating that voluntary medical male circumcision (VMMC) is an effective method to reduce men’s chances of acquiring HIV, use of the procedure has been advocated in Kenya. Media messages shape popular understandings of the benefits and limitations of male circumcision. The objectives of this study were to (1) investigate promotion messages in a popular online newspaper to determine how the limitations of male circumcision are represented, and whether condom use is still being promoted; and (2) gain insight into popular understandings of the limitations of this new procedure through newspaper reader comments.

**Methods:**

A content analysis was conducted on 34 online media articles published by the Daily Nation between January 1, 2008 and December 31, 2010. Information about condom promotion, partial immunity, limitations and complications of the procedure, as well as emergent themes, were analyzed.

**Results:**

Results demonstrated an irregular and occasionally misleading presentation of these topics and a perceived lack of objective information about the risks and limitations of VMMC.

**Conclusions:**

There is a need for governmental and non-governmental public health organizations to engage with the media to improve risk messaging.

## Background

The World Health Organization (WHO) and the Joint United Nations Programme on HIV/AIDS (UNAIDS) estimated that in 2009, 33.3 million individuals worldwide were infected with HIV [1]. Many live in sub-Saharan Africa, where there were an estimated 1.3 million AIDS related deaths in 2009 [1]. Since 1987, researchers have been attempting to develop a vaccine to curb HIV transmission; however, progress has been limited. As such, many researchers have directed their efforts to investigating other methods to prevent HIV transmission, including microbicides and adult voluntary medical male circumcision (VMMC).

The results of three clinical trials in sub-Saharan Africa indicate that male circumcision may provide a protective effect of approximately 60% against the acquisition of HIV in heterosexual males [2-4]. In 2007, WHO and UNAIDS announced their support of VMMC as an effective HIV prevention method and recommended its adoption as an additional strategy to combat heterosexual HIV transmission [5]. In response, the Government of Kenya launched the Voluntary Medical Male Circumcision Programme in November 2008 [6]. One objective of the program is to circumcise approximately 80% of uncircumcised men aged 15 to 49 [6].

VMMC is supported by several international organizations, prominent researchers, and the Kenyan government; however, debate remains concerning its promotion. Male circumcision is practiced among certain ethnic groups in Kenya, where it is traditionally tied to a cultural rite of passage and aids in defining ethnic identity boundaries. Male circumcision does not offer full protection against HIV infection. Concerns have also been raised that partially protective HIV prevention methods might be perceived as fully protective, which could lead to sexual disinhibition or a reduction in condom use [7]. In one study among young South African adults, male participants thought that, if vaccinated with a partially effective vaccine, they could halt other HIV prevention strategies, such as using condoms [7]. It is critical that messaging about VMMC be informative, accurate, and culturally sensitive [5] and emphasize that VMMC does not afford full protection against HIV infection [8].

The Kenyan Ministry of Public Health & Sanitation has developed a VMMC Communication Guide, which includes the utilization of the media to promote “frequent and accurate coverage of VMMC in Kenya” [9]. Mass media (e.g. radio, television, cinema, newspapers, pamphlets, and posters) provide an opportunity to communicate to the general public. For many people, their knowledge, attitudes, and beliefs about a topic or event are shaped, in part, by media framing. For example, the attractiveness of a vaccine is affected by whether its efficacy is portrayed as a probability or a ratio [10]. Moreover, studies demonstrate that individuals make decisions about medical procedures based on potential losses and gains [11-13]. This is further compounded by the news media’s use of empirical observations involving numbers [14]. Peters and colleagues have determined that individuals with low numeracy, the ability to understand and interpret numbers, are influenced by number formatting, have increased risk perceptions when presented with medical information using frequencies, and are more likely to be influenced by message framing [15-17]. Most lay individuals do not understand quantitative measures of risk; instead, people tend to understand qualitative statements about risk in the media [18]. Another important factor influencing risk understanding may be the placement of risk statements within the media article itself. It can be argued that, statements placed at the beginning of a media story impart greater importance and weight based on the assumption that individuals are most likely to read the opening paragraphs or story lead [19]. Therefore, the way in which the limitations of VMMC and sustained condom use are portrayed in the media may have an impact on individual choices and understandings.

Mass media campaigns promoting HIV prevention methods can be an effective HIV prevention tool [20], and have been shown to be one of the main sources of HIV/AIDS information for the general population in African countries [21-23]. Among participants of a study in Ghana, 76% of women and 84% of men considered media to be their main source of HIV/AIDS knowledge [21].

In light of the Phase III population level vaccine trial completed in Thailand in 2009, which only showed moderately efficacious results, it is important to understand how the media are communicating the reality of partial protection, and how individuals may understand partial protection risk messaging when, or if, it is communicated [24]. In this sense, the portrayal of VMMC in the media may be a useful case study for communication strategies of potential future HIV prevention campaigns. To our knowledge, only one other study has investigated newspaper and magazine articles published about VMMC as an HIV prevention method [25]. However, it did not include newspapers from Kenya.

Given the current push to promote VMMC, and the importance of monitoring VMMC promotion messages, this research has two primary objectives. First, to investigate VMMC promotion messages in a popular Kenyan newspaper to determine how the limitations (partial protection) of VMMC are represented, and whether condom use is still being promoted. Secondly, this study seeks to explore public understandings of the limitations of VMMC in Kenya, through online newspaper reader comments.

## Methods

### Data source

Founded in Kenya in 1959, Daily Nation is the largest independent newspaper in East and Central Africa, and is published by Nation Media Group (NMG) Limited [26]. The Daily Nation was selected due to its ease of access for the study team and large readership in both electronic and print newspapers. It is estimated that in 2006 the Daily Nation had a daily print circulation of 185,000 copies [27], and in 2011 the Daily Nation website was the most popular Kenyan website and the 10^th^ most popular website overall in Kenya [28].

### Data collection

Newspaper articles were searched using the Daily Nation website search tool, as the popular media archives LexisNexis® and Factiva® do not index any Nation Media Group material. The website was searched using the following keyword terms: ‘male cut’; ‘male circumcision’; ‘circumcision’; ‘penis’; and ‘foreskin’. The inclusion criteria required that at least 60% of the article focus on VMMC programs or clinical trials as a method to prevent HIV infection. Following the practice of other researchers in the field, it is important that decisional and definitional rules are established in advance [29].

Following this sampling framework, a total of 34 online media articles published during a two-year period between January 1, 2008 and December 31, 2010 were selected for final coding and analysis. This period coincided with the launch and expansion of the Kenyan VMMC programme. Utilizing electronic media also enabled an exploration into readers’ understanding of promotion messages through analysis of reader comments.

### Data analysis

A qualitative thematic analysis was conducted on the selected online media stories published by the Daily Nation. Themes regarding the portrayal of this information were then compared to existing literature about framing [30]. Framing is “the process of culling a few elements of perceived reality and assembling a narrative that highlights connections among them to promote a particular interpretation” [31]. Thus, framing works at multiple levels. At the macrolevel, framing serves to present a topic, whereas at the microlevel, framing may influence how individuals read and interpret the information provided to them [32]. Prior to in-depth analysis the articles were categorized in two groups; Op/Ed, and News, to reflect the differences in types and authorship of the articles. Articles in the Op/Ed category included opinion, editorials, and letters, which can be submitted by anyone online to the Daily Nation. Monetary compensation is not provided to individuals with successful submissions, thus all individuals including employees and members of the public (e.g. academic researchers, lay individuals in the community) may author an Op/Ed article. News articles were considered to be all other articles written by paid employees of the Daily Nation. Though both Op/Ed and news articles are reviewed by editors before publication, articles written by members of the public may provide a different stance on a topic compared to those presented in traditional news articles. A previous study investigating reader comments suggests that online comments can be a valuable and reliable source of data to investigate individuals’ understandings about health crisis events [33].

Articles were analyzed for statement placement (beginning, middle, or end) of key phrases about limitations and condom promotion. As news stories are typically constructed with the main points or conclusions at the beginning and work backwards to the elements contributing to that lead throughout the full article, examining where prevention messages are situated in a media text is important. In this case, a statement was defined as a written communication of particulars or facts, often no more than a sentence [34]. To operationalize what constitutes the beginning, middle and end of newspaper articles, the number of paragraphs in each were counted and divided in to three equal parts; with the first third considered the ‘beginning’, the second third the ‘middle’, and the last third the ‘end’. If present, summary sections located before the main text were also included in the first third of the article. Following previous studies, statement placements located at the beginning of a story were assigned a greater weight, followed by statements located at the end, and then statements placed at the middle [35]. This importance in placement operates on the assumption that readers typically scan the beginning of a story, may skip to the end if they are moderately interested, and will only read the entire text if they are very interested in the topic [35]. A distinction was also made between explicit and non-explicit statements. To be explicit, a statement had to be clearly written in plain language, leaving nothing implied about a specific fact or suggestion. Idioms were considered to be non-explicit statements.

Articles were systematically coded and analyzed using NVivo9. Open coding was first used to identify if the articles as a whole supported or refuted VMMC. A second round of coding was used to specifically identify how the limitations of male circumcision as an HIV prevention method were communicated, and if condom promotion was included in the messages to reinforce that male circumcision does not fully protect against HIV infection. In addition to the published articles, reader comments were analyzed for uptake and understanding of the media messages.

### Validity and reliability

As only one individual coded and analyzed the newspaper stories, no inter-coder reliability score was produced. To ensure validity, an audit trail was maintained. As part of our reflexive process, frequent discussions were held with team members to develop conceptual categories, ensure that the analysis was not prematurely closed and to provide an opportunity to challenge emerging patterns in the data. Moreover, an external reviewer not familiar with the study also read the results to ensure trustworthiness.

## Results

**Figure 1:**
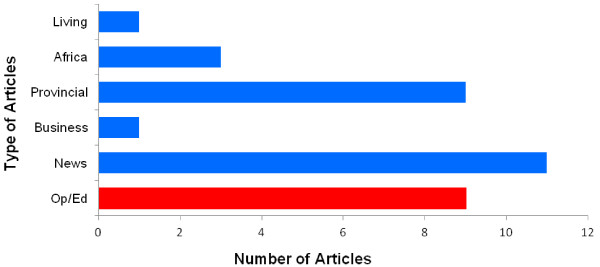
**Type of articles included in the final analysis.** Types of articles collected from the *Daily Nation* online newspaper and included in the final analysis. All articles types, except Op/Ed articles (red), were categorized as a type of news story (blue). The sections “Living,” “Africa,” “Provincial,” “Business,” and “News” are how the Daily Nation organizes its news articles on their website using headings accessed on the homepage.

Of the 34 articles included in the final analysis, 9 were Op/Ed letters or opinion articles published in the online Daily Nation newspaper (Figure 1). Fifty-six percent of the articles were published in 2008, with a significant cluster (n = 14) occurring in September, October, and November. This period reflects the time in which the Kenyan Government was preparing to begin its VMMC programme.

### Portrayal of the limitations of male circumcision – news articles

One article was published to “warn” individuals that male circumcision does not fully prevent HIV infections, and indicated that it was released due to a surge in individuals’ seeking VMMC at medical facilities. The title, “Cut will not give HIV immunity”, clearly communicated the limitation of VMMC. Other than this article, only two of the 24 remaining news articles published by the Daily Nation explicitly indicated that VMMC does not fully protect against HIV infection in males who obtain the procedure. Only one article indicated that females are not protected from HIV if their male partners were circumcised. The limitations, when explicitly identified, were communicated in a way which people were being remonstrated or warned. An example of such statements is provided below:

"“…it should be understood that male medical circumcision does not make one immune to the virus… No one should think they now have a license not to use a condom or go for HIV testing.” (March 10, 2009) "

These messages appeared to be concerned with elevating public awareness and reminding readers in an indirect way that VMMC does not fully protect against HIV infection, while the tone implies that people should ‘know better’.

A number of articles (n = 17) referenced the three published clinical trials to indicate the approximate percentage of risk reduction afforded by obtaining VMMC. In some news stories, two or more variations of the same statement were reiterated in different parts of the news story. In the vast majority of cases, this was expressed as an advantage of male circumcision and not in a manner that would alert individuals to the partial protection afforded by VMMC. These percentages technically indicate that VMMC only affords partial immunity; however, the framing of the article camouflages VMMC’s limitations. The inconsistency of the terminology used may be difficult for lay people to understand and thus may not be conducive to fully understanding the degree of protection VMMC provides against HIV. Moreover, some phrasing made it difficult to determine if VMMC protects the individual that has the procedure or their sexual partner. To add to the confusion, not all reported risk estimates were the same, with some articles reporting 50%, 53%, 60%, 61%, or 66% reduction in risk. Also, when individuals were not provided with denominator information to provide context, it can cause further confusion about perceived risk [36]. For example, a 50% reduction can reduce one’s chance of infection or transmission from a ½ chance to a ¼ chance, or 1/100,000 chance to 1/50,000. Denominator information helps explain relative risk reduction of becoming infected, and helps answer the question what does a 50% reduction really mean for an individual? Qualifying the statement with denominator data gives individuals additional information about their actual relative risk. Denominators were not utilized and the limitations of VMMC were camouflaged, as demonstrated in the following quotes:

"“…male circumcision can reduce the rate of HIV infections in heterosexual men by 50 per cent.” (June 23, 2008)"

"“…male circumcision cuts the risk of HIV transmission by 60 per cent.” (February 24, 2009) "

The words limit, limits, limitation, or partial protection were never used in the news articles to describe the partial protection afforded by VMMC. While no direct complications were listed or mentioned to be associated with the procedure itself, a few statements were made indicating that engaging in premature sexual intercourse after having the procedure would increase individuals’ chances of infection due to tearing of unhealed skin. It was not clear if the statements were referring to bacterial or HIV infection.

### Portrayal of the limitations of male circumcision – Op/Ed articles

Unlike news articles, Op/Ed articles focused on the limitations and perceived risks of adopting VMMC as an HIV prevention strategy. Though only two articles used the words “partial protection” when referring to limitations of VMMC, they often expressed views that media should do more to communicate the limitations of VMMC to the general public as illustrated by the following:

"“Research did not indicate males get HIV infection because they are uncircumcised, nor that circumcised males are immune to the virus. I believe the Press has a moral duty to bring out this truth.” (September 28, 2008)"

"“…linking mass male circumcision to the Aids (sic) fight without proper information perpetuates the fallacy that it offers total protection.” (December 6, 2009)"

### Promotion of other HIV prevention methods – news articles

As was the case when articles mentioned partial protection, most news articles did not explicitly encourage consistent and sustained condom use, or how it should be used in conjunction with other methods. Some of the articles indicated that future VMMC promotion programs should or would emphasize condom use, or indicated that the WHO strongly encouraged all HIV prevention strategies should include the promotion of condom use. This was often reported as direct quotes from experts in the field, and did not seem to be a focus of the article. Condom promotion was usually included in sentences that also indicated the need for abstinence, counselling, and/or education. Three articles, including one Op/Ed article, specifically mentioned abstinence as another method that should be emphasized to prevent HIV infection. One of the quotes is as follows:

"“He [National AIDS & STI’s Control Programme Director, Nicholas Muraguri] said men still had to follow other protective measures such as avoiding multiple sexual partners and using condoms.” (February 24, 2009)"

### Promotion of other HIV prevention methods – Op/Ed articles

The opinion pieces published in the online newspaper indicate that at least some individuals are concerned with the perceived lack of emphasis on supplementing VMMC with other prevention methods. The majority of Op/Ed articles expressed the same concern for misinterpretation of facts or information by the general public as summarized in the following representative quote:

"“In my view, the promotion of male circumcision should be accompanied by riders encouraging people to use other means of protection even after the cut. Otherwise we run the risk of creating the false impression that once a man is circumcised, he can start sleeping around without any care in this world.” (September 29, 2008)"

### Statement placements

**Figure 2:**
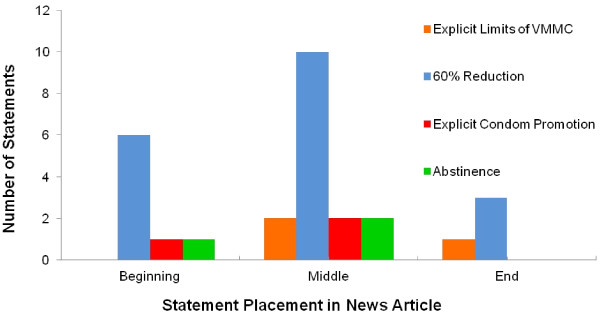
**Statement Placements.** Placement of statements, communicating the limitations of VMMC, promoting condom use and abstinence, in news articles from the online *Daily Nation* newspaper.

The limitations of VMMC was predominantly communicated through numeric statements regarding the reductions in HIV infection rates, as opposed to keeping in line with corresponding public health messaging that additional precautionary acts (i.e. condom use) were required. A total of twenty statements about the 60% reduction were made, of which only seven were included in one of the most prominent sections of a news story, the first third of the article (Figure 2). If these “60% reduction” statements are excluded, there were no explicit statements communicating the limits of VMMC at the beginning of a news story. Out of the three statements that directly address the limitations of VMMC for HIV prevention, two were found in the middle of the news story and one at the end. Their placement thus de-emphasizes the importance of the limitations and reduces the likelihood of their being read. Similarly, the vast majority of statements promoting abstinence or condom use were located in the middle of the news stories, with only one explicit mention of condom promotion at the beginning of an article. Overall, the uptake of this important information by the reader might be limited due to its placement within the article.

### Reader comments and perspectives of male circumcision

Several comments expressed concern over “other people” not understanding that VMMC does not afford full protection. One reader commented on the frequently used phrase of “60 per cent risk reduction,” indicting that it was hard to interpret its meaning, and identified it as a failure of effective risk communication.

"“This was a fatal error in communicating science. The scientist who found out this should have devised a communication strategy of the results understandable to the common man. Even us technology suffy [savvy] readers, just try to figure out what it means you ...60 percent reduction to a lay man may mean having sex with partners that do not exceed 60 and you will be safe…” (December 6, 2009) "

Some questions were raised as to why male circumcision is being promoted aggressively when abstinence and condom use afford better protection from HIV transmission. Some commentators were clearly able to identify the limitations of VMMC and thus seemed to be questioning why it was being promoted over other methods. The comment “…they [the public] should be fully informed of the risks and of the nature of the benefits - including the fact that they [benefits] can all be achieved without amputating part of the penis.” (September 7, 2008) exemplifies that alternative, yet highly effective, methods to reduce one’s chances of getting infected with HIV seem to be downplayed in the VMMC promotion campaign. Another example of such a quote is:

"“We now have people calling circumcision a "vaccine" or "invisible condom", and viewing circumcision as an alternative to condoms. ABC [Abstinence, Be faithful, Condoms] is the way forward.” (April 6, 2010)"

## Discussion

The partial or incomplete presentation and assessment of information from news articles regardless of its pertinence or accuracy, can negatively affect people’s understandings, opinions, and decisions related to the topic [37]. The results of this analysis demonstrate that, in the Daily Nation newspaper, the limitations of VMCC, its possible negative consequences and importance of condom use is not being communicated regularly or explicitly in individual published news stories.

The few times that the limitations and potential consequences of VMMC were explicitly communicated, newspaper articles seemed to present information about the limitations of VMMC by camouflaging limitations by portraying them as benefits to identify potential drawbacks of VMMC. The limitations of VMMC as an HIV prevention method were camouflaged in positive language, which highlighted risk reduction and not the fact that VMMC does not afford full protection. Even if a procedure reduces the risk of infection, it does not mean that the risk of becoming infected with HIV is eliminated. Previous research has indicated that for health promotion messaging, “gain” framed messages, where the benefits of a procedure or action are emphasized make individuals more receptive to undertake the procedure or behaviour than approaches that emphasize a loss frame [38]. While this suggests it is logical to present VMMC in a “gain frame” in order to encourage people to receive the procedure [38], it should not necessarily eliminate the need for further communication about individuals’ continued susceptibility to HIV. Other articles blamed the general population for not understanding the limitations of VMMC, using statements such as “it should be understood that” and “residents have been warned”.

The use of percentages to communicate both the benefits and limitations of VMMC may be problematic and potentially misleading, as most lay people are not numerically literate and may misinterpret the meaning [39,40]. Health numeracy is an important ability that helps people understand relative risks and benefits from medical procedures [14]. Research has consistently shown that vulnerable groups, including those who live in poverty, often have low numeracy [41], with those with lower numeracy tending to be “less sensitive to numerical differences in probability” [42]. One reader’s comment directly addressed this, writing that even he, a “technology suffy [savvy] reader” has difficulty understanding its meaning. There is also evidence that the context of the presentation of results is just as important as the numerical format in which results are presented [40].

None of the messages communicated that while VMMC may help reduce the numbers of HIV infection in a total population, a 60% reduction is not a highly effective method of protecting oneself from HIV infection, given the availability of other highly effective prevention methods. The absence of specific information reporting, in this case not using the words “limit” or “limitation” is an important finding, as it seems to contradict the recommendations put forth by the WHO and other experts to continually identify not only the benefits but also the limitations of VMMC in their risk communication strategies.

The majority of the 25 news articles did not emphasize the need for additional protective measures against HIV. Previous research has shown that the media can be very effective in communicating about HIV prevention methods. One study found that people were less able to identify abstinence as an HIV prevention method because it was the least likely method reported in the media [21]. This may suggest that if VMMC and condom promotion are not communicated regularly in the same articles, people may not associate VMMC with the need for continued condom use even if condom use is still promoted in other news articles.

In one study investigating global newspaper reporting of VMMC trials, of the 219 articles found 82 articles reported that individuals should use condoms [25], though there is no information on how this information was reported. The results from the current study, however, indicate, the Daily Nation, does not follow the same worldwide pattern in promoting condom use. This suggests that the individuals in Kenya are not receiving critical information about the limitations of VMMC as often as they could and should be.

While the news articles did not include the limitations of VMMC or promote sustained condom use, the Op/Ed articles often emphasized these points. While news articles are based on facts gathered by reporters, Op/Ed articles are the opinions of an individual. Thus, Op/Ed articles may be more likely to express views different than the newspaper editors. Op/Ed articles included evidence about the limitations of VMMC and the importance of condom use, and expressed concern over their perceived lack of prominence in the VMMC programme. Few articles expressed a strong sentiment against VMMC. Rather the focus was on the need for more public education, or what some of the articles referred to as “enlightenment”. Even though these critical messages are present in Op/Ed articles, it is unclear how many people read this type of article, and if individuals assign to them the same importance as news articles.

Another important finding was the placement of important messaging within the news stories. The lack of statements located at the beginning of the articles, other than the 60% phrase, communicating the limitations of male circumcision is telling. Little emphasis was placed on communicating limitations in these news stories. The relatively few statements encouraging other methods to prevent HIV transmission, such as abstinence and condom use, were not emphasized. Their placement, almost always within the body or middle of the news story, may indicate that few people read these important messages.

Though the strength of this research lies in the interpretation of the messages provided by the newspaper articles, readers’ comments provide some insight into understanding and concerns regarding VMMC. Similarly to the Op/Ed articles, many individuals wrote comments referring to the benefits and limitations of VMMC, the need for sustained condom promotion, and expressed concern that the media and government should provide better and more comprehensive risk communication.

### Recommendations

This research demonstrates that one Kenyan newspaper, the Daily Nation, is not effectively or comprehensively communicating the limitations of VMMC. Thus, several recommendations are being proposed to improve risk communication. First, the government could provide regular news releases or information sessions to the media, which communicate the procedures’ partial protection. This information should be presented in a clear and concise manner, without the use of probabilities. This may be challenging, as the VMMC campaign may no longer be considered newsworthy of reporting. Second, continued monitoring is needed to ensure media messaging concerning VMMC includes discussion of limitations and encourages condom use following circumcision. If this emphasis does decline, this should signal the need for a repeated media campaign on the part of public health professionals to raise public awareness. Third, further research should be conducted to investigate whether the general public understands that VMMC does not completely protect them from HIV infection and the importance of sustained condom use whether or not they are circumcised. This research could involve surveys of people’s attitudes or employ more qualitative methods of focus group discussions and interviews.

### Limitations

This study has several limitations. The Daily Nation online newspaper was selected due to its ease of access and wide readership across Kenya, however no other newspapers were selected. It is possible that many Kenyans prefer other news outlets to receive information, and that other newspapers may communicate the limits of VMMC and encourage sustained condom promotion in a more pronounced way. The Daily Nation is also an English language newspaper. Even though many Kenyans speak English, it is possible that non-English newspapers may represent VMMC in a different way. This small sample size is therefore only a small portion of a larger news media landscape. As Daily Nation is not indexed by any news database system, it is possible that additional articles may have been missed. Moreover, newspapers are only one source of mass media that shapes public understandings of risk information. NGO and Government pamphlets and community outreach programs may communicate slightly different messages than that from newspapers because newspapers are independent bodies that present information in ways designed to attract increased readership. In addition no inter-coder reliability score was produced as only one individual coded and analyzed the data, though a group reflexive process and audit trail were employed. The single coder developed conceptual categories, including operationalized definitions, collaboratively with a senior-member of the research team, who also served as auditor of the interpretive coding. While double coding was not undertaken, emerging interpretations were challenged to ensure against premature closure of ideas. Lastly, in September 2011, Kenya was cited as the leading country in the uptake of VMMC in Africa, thus a limitation is the time period in which this analysis was conducted (2008–2010) because it does not include the 2011 calendar year [43].

## Conclusions

This analysis demonstrated that newspaper articles from the Daily Nation fail to emphasize the importance of understanding the limitations of VMMC and do not promote the sustained use of condoms in the majority of articles published regarding VMMC. This finding seems to contradict the public health recommendations put forth by the WHO and many other researchers.

The absence of certain forms of information in newspapers can be a crucial component in understanding public health risk communication. As a large number of individuals receive the majority of their information about HIV/AIDS from the news media, it is important that the news media communicate clearly and do not omit critical information. The risk of still acquiring HIV after circumcision may be more effectively presented using words, rather than numerical values. As Kenya continues to have a high VMMC uptake, it is important that articles focus not only on its potential to reduce HIV infections, but should also explicitly communicate the limitations of the procedure and remind individuals that condom use remains essential.

## Competing interests

The authors have no competing interests to declare.

## Authors’ contributions

CNM helped design the study, collected data, conducted the data analysis and interpretation, and wrote the manuscript. RL conceived the study, designed the study, participated in data analysis and interpretation, and manuscript preparation. SMD worked closely alongside CNM throughout the design phase, the data analysis and interpretation, serving as auditor in this process, and in manuscript preparation. LHT was involved in study design, and manuscript preparation. AEB contributed to manuscript preparation. All authors read and approved the final manuscript.

## Pre-publication history

The pre-publication history for this paper can be accessed here:

http://www.biomedcentral.com/1471-2458/12/465/prepub

## References

[B1] Joint United Nations Programme on HIV/AIDS (UNAIDS) AIDS scorecards: overview: UNAIDS report on the global AIDS epidemic 2010 2010 4

[B2] Bailey RC Moses S Parker CB Agot K Maclean L Krieger JN Williams CF Male circumcision for HIV prevention in young men in Kisumu, Kenya: a randomized controlled trial Lancet 2007 269 9562 643 656 17321310 10.1016/S0140-6736(07)60312-2

[B3] Auvert B Taljiarred D Lagarde E Sobngwi-Tambekou J Sitta R Puren A Randomized, controlled intervention trial of male circumcision for reduction of HIV infection risk: The ANRS 1265 Trial PLoS Med 2005 2 11 1112 1122 10.1371/journal.pmed.0020298 PMC1262556 16231970

[B4] Gray RH Kigozi G Serwadda D Makumbi F Watya S Nalugoda F Kiwanuka N Moulton LH Chaudhary MA Chen MZ Sewankambo NK Wabwire-Mangen F Bacon MC Williams CFM Opendi P Reynolds S Laeyendecker O Quinn TC Wawer MJ Male circumcision for HIV prevention in Rakai, Uganda: A randomised trial Lancet 2007 369 9562 658 666 10.1016/S0140-6736(07)60313-4 17321311

[B5] World Health Organization (WHO) Joint United Nations Programme on HIV/AIDS (UNAIDS) New data on male circumcision and HIV prevention: Policy and programme implications 2007 3 10

[B6] Government of Kenya, Ministry of Public Health and Sanitation National AIDS and STI Control Programme: Progress report on Kenya's voluntary medical male circumcision programme, 2008–2009: Summary 2010 1 14

[B7] Sayles J Macphail CL Newman PA Cunningham WE Future vaccine acceptability among young adults in South Africa Health Educ Behav 2010 37 193 210 10.1177/1090198109335654 19509123 PMC2866130

[B8] Rennie S Muula AS Westreich D Male circumcision and HIV prevention: Ethical, medical and public health tradeoffs in low-income countries J Med Ethics 2007 33 357 361 10.1136/jme.2006.019901 17526688 PMC2598273

[B9] Government of Kenya, Ministry of Public Health and Sanitation Voluntary Medical male circumcision (VMMC) communication guide for Nyanza Province 2010

[B10] Slovic P Fischhoff B Lichtenstein S Why study risk perceptions? Risk Anal 1982 2 83 93 10.1111/j.1539-6924.1982.tb01369.x

[B11] Kahneman D Tversky A Choices, values, and frames Am Psychol 1984 39 4 341 350

[B12] Entman RM Framing: Toward clarification of a fractured paradigm J Commun 1993 43 4 51 58 10.1111/j.1460-2466.1993.tb01304.x

[B13] Garcia-Retamero R Galesic M How to reduce the effect of framing on messages about health J Gen Intern Med 2010 25 12 1323 1329 10.1007/s11606-010-1484-9 20737295 PMC2988162

[B14] Ancker JS Kaufman D Rethinking health numeracy: A multidisciplinary literature review J Am Med Inform Assn 2007 14 713 721 10.1197/jamia.M2464 PMC2213486 17712082

[B15] Peters E Hart S Fraenkel L Informing patients: The influence of numeracy, framing, and format of side effect information on risk perceptions Med Decis Making 2011 31 432 436 10.1177/0272989X10391672 21191122

[B16] Peters E Levin IP Dissecting the risky-choice framing effect: Numeracy as an individual-difference factor in weighting risky and riskless options Judgm Decis Mak 2008 3 435 438

[B17] Peters E Västfjäll D Slovic P Mertz CK Mazzocco K Dickert S Numeracy and decision making Psychol Sci 2006 17 407 413 10.1111/j.1467-9280.2006.01720.x 16683928

[B18] Wahlberg AA Sjoberg L Risk perception and the media J Risk Res 2000 3 1 31 50 10.1080/136698700376699

[B19] Driedger SM Creating shared realities through communication: Exploring the agenda-building role of the media and its sources in the E. coli contamination of a Canadian public drinking water supply J Risk Res 2008 11 1–2) 23 40

[B20] Palmgreen P Noar SM Zimmerman RS Edgar T, Noar S, Freimuth VS Mass media campaigns as a tool for HIV prevention Communication Perspectives on HIV/AIDS for the 21st Century 2007 1 Routledge, New York 221 252

[B21] Benefo KD Takyi BK Mass media effects on AIDS knowledge and sexual behaviour in Africa with special reference to Ghana Int J Sociol Soc Pol 2002 22 4–6 77 99

[B22] Muthivhl E Sodi T Maunganidze L Mudhovozi P Knowledge, attitudes and perceptions of HIV and AIDS by rural South African secondary school learners J Psychol Africa 2011 21 4

[B23] HSRC South African national HIV prevalence, incidence, behaviour and communication Survey 2008: Turning the tide among teenagers?

[B24] Rerks-Ngarm S Pitisuttithum P Nitayaphan S Kaewkungwal J Chiu J Vaccination with ALVAC and AIDSVAX to prevent HIV-1 infection in Thailand New Engl J Med 2009 361 2209 2220 10.1056/NEJMoa0908492 19843557

[B25] Wang AL Duke W Schmid GP Print media reporting of male circumcision for preventing HIV infection in sub-Saharan Africa B World Health Organ 2009 87 8 10.2471/BLT.09.030109 PMC2733269 19705009

[B26] About Daily Nationhttp://www.nation.co.ke/meta/-/1194/1172/-/ojmv8c/-/index.html

[B27] Onyebadi U Oyedeji T Newspaper coverage of post political election violence in Africa: An assessment of the Kenyan example Media, War & Conflict 2011 4 3 215 230 10.1177/1750635211420768

[B28] Mghendi T Kenya internet users - which sites do they visit. TiEL ICT Blog

[B29] Sampert J Trimble L Appendix: A primer on content and discourse analysis methodology Mediating Canadian Politics 2009 1 Pearson Education Canada, Toronto

[B30] De Vreese CH News framing: Theory and typology IDJ 2005 13 1 51 62

[B31] Entman RM Framing bias: Media in the distribution of power J Commun 2007 57 163 173 10.1111/j.1460-2466.2006.00336.x

[B32] Scheufele DA Tewksbury D Framing, agenda setting, and priming: The evolution of three media effects models J Commun 2007 57 9 20

[B33] Henrich N Holmes B What the public was saying about the H1N1 vaccine: Perceptions and issues discussed in on-line comments during the 2009 H1N1 pandemic PLoS One 2011 6 4 e18479 10.1371/journal.pone.0018479 21533161 PMC3078916

[B34] Altheide DL Creating fear: News and the construction of a crisis: New York 2002 Aldine De Gruyter, NY

[B35] Driedger SM Jardine C Boyd A Bhavnita M Do the first 10 days equal a year? Comparing two Canadian public health risk events using the national media Health Risk Soc 2009 11 1 39 53

[B36] Roch JP Muskavitch MAT Limited precision in print media communication of West Nile virus risks Sci Commun 2003 24 3 353 365 10.1177/1075547002250300

[B37] Ndiwane AN Community health, media, and policy in Sub-Saharan Africa: A primary prevention approach to the AIDS crisis ABNF J 2000 11 4 83 87 11760309

[B38] Rothman AJ Salvoey P Shaping perceptions to motivate health behavior: The role of message framing Psychol Bull 1997 121 1 3 19 9000890 10.1037/0033-2909.121.1.3

[B39] Fagerlin A Zikmund-Fisher BJ Ubel PA Helping patients decide: Ten steps to better risk communication JNCI 2011 103 19 1436 1443 10.1093/jnci/djr318 21931068 PMC3218625

[B40] Visschers VHM Meertens RM Passchier WWF de Vries NNK Probability information in risk communication: A review of the research literature Risk Anal 2009 29 2 267 287 10.1111/j.1539-6924.2008.01137.x 19000070

[B41] Dieckmann NF Slovic P Peters E The use of narrative evidence and explicit likelihood by decision makers varying in numeracy Risk Anal 2009 10 1473 1488 19671102 10.1111/j.1539-6924.2009.01279.x PMC7159394

[B42] Reyna VF Nelson WL Han PK Dieckmann NF How numeracy influences risk comprehension and medical decision making Psychol Bull 2009 135 6 943 973 19883143 10.1037/a0017327 PMC2844786

[B43] National AIDS Control Councilhttp://www.nacc.or.ke/index.php?option= com_content&view=article&id=250:kenya-leads-in-voluntary-medical-male-circumcision&catid=107:news&Itemid=174

